# Unraveling the Network Signatures of Oncogenicity in Virus–Human Protein–Protein Interactions

**DOI:** 10.3390/e27121248

**Published:** 2025-12-11

**Authors:** Francesco Zambelli, Vera Pancaldi, Manlio De Domenico

**Affiliations:** 1 Department of Physics and Astronomy “Galileo Galilei”, University of Padova, 35121 Padova, Italy; 2 Centre de Recherches en Cancérologie de Toulouse (CRCT), Université de Toulouse, Inserm, CNRS, 31100 Toulouse, France; 3 Padua Center for Network Medicine, University of Padova, 35131 Padova, Italy; 4 Istituto Nazionale di Fisica Nucleare Sezione di Padova, 35131 Padova, Italy

**Keywords:** network medicine, multilayer networks, oncogenic viruses

## Abstract

Background: Climate change, urbanization, and global mobility increase the risk of emerging infectious diseases with pandemic potential. There is a need for rapid methods that can assess their long-term effects on human health. In silico approaches are particularly suited to study processes that may manifest years later, under the assumption that perturbed biomolecular interactions underlie these outcomes. Here we focus on viral oncogenicity—the ability of viruses to increase cancer risk—which accounts for about 15% of global cancer cases. Methods: We characterize viruses through multilayer representations of protein–protein interaction (PPI) networks reconstructed from the human interactome. Statistical analyses of topological features, combined with interpretable machine learning models, are used to distinguish oncogenic from non-oncogenic viruses and to identify proteins with potential central role in these processes. Results: Our analysis reveals clear statistical differences between the network properties of oncogenic and non-oncogenic viruses. Furthermore, the machine learning approach enables classification of virus–host interaction networks and identification of relevant subsets of proteins associated with oncogenesis. Functional enrichment analysis highlights mechanisms related to viral oncogenicity, including chromatin structure and other processes linked to cancer development. Conclusions: This framework enables virus classification and highlights mechanisms underlying viral oncogenicity, providing a foundation for investigating long-term health effects of emerging pathogens.

## 1. Introduction

In the last years, the profound impact of viral pathogens on human health has become unmistakably evident, with the COVID-19 global pandemic serving as a stark reminder of their relevance. Beyond the acute phase of an infection, it is increasingly recognized that certain viral pathogens can cause persistent health issues [1,2,3,4], notably among them, the potential for oncogenicity [5,6,7,8,9], which poses a substantial and multifaceted challenge for both individuals and public health. Such a risk is further emphasized by recent discoveries about cellular mechanisms that lead to DNA degradation and cell senescence subsequent to SARS-CoV-2 infection [10].

Over the last two decades, the study of complex networks has undergone significant advancements, which have broadened its impact on various fields. One of the major breakthroughs concerns the application of complex network theory to systems biology and medicine, leading to network medicine [11], an interdisciplinary field that enables studying diseases and their underlying mechanisms in terms of network structure and dynamics [11,12,13,14]. This approach focuses on understanding phenotype in terms of the interdependence between proteins, genes, metabolites, biological processes and drugs, providing a suitable framework to build in silico models, shedding light on the underlying biological systems and on complex disease–disease interactions [13,15,16].

One of the core elements used in network medicine are protein–protein interaction (PPI) networks [17,18,19,20,21], models able to represent the interactions between proteins in a biological system. These interactions can be physical (direct) or functional (indirect) and are crucial for a variety cellular processes and functions [22,23]. PPI networks provide a way to visualize and study how proteins interact with each other, forming complex relationships within a cell or organism.

Recent work exploited such tools to gain deeper knowledge about the SARS-CoV-2 virus [24,25,26,27], for example allowing integration of interaction between proteins, symptoms and biological pathways to devise drug repurposing strategies [18,28].

Considering the increasing number of results obtained from the study of complex diseases by means of PPI networks, we ask whether it is possible to apply such methods to determine the potential oncogenicity of a virus following virus–host interactions. The wide availability of this type of PPI data [22] offers a a valid starting point to tackle this challenge.

Moreover, the prospect of utilizing online data and mathematical methodologies to establish a classification system for viruses based on their oncogenic potential holds significant promise for systems medicine. In fact, the outcome of transparent computational models, based on network analysis, can not only serve as an invaluable springboard for ongoing clinical and medical investigations, but also provide valuable insights into the effects of emergent viruses, which have yet to undergo comprehensive scrutiny due to their relatively short existence and the lack of longitudinal data. A salient case in point is the SARS-CoV-2 virus, for which evidence about possible long term effects on human organs and systems are already under investigation [29,30,31]. In this case, a complete examination of the whole spectrum of its long-term effects from a clinical perspective might require at least a decade.

To address this challenge, we use a multilayer framework [32,33] where multiple virus–host PPI networks are coupled to build multiplex networks [34,35]. Systems consisting of multiple interacting networks have attracted considerable attention, owing to the discovery of novel structural and dynamical features in coupled cases that differ from those observed in uncoupled representations of the same system, as reported for transportation [36], urban [37], ecological [38], social [39], financial [40], and many other systems [34,41,42,43,44], to mention some emblematic examples (see [33] for a recent review).

Here, we use multilayer network models to construct a holistic system able to account for perturbations due to distinct external agents (i.e., viruses) to the same target (i.e., human cells). Considering the whole human interactome as a proxy for this target, each layer can be understood as a description of the interaction with a specific virus.

In fact, this framework facilitates the identification of commonalities and shared properties between these distinct layers of interaction with the human proteins, being suitable to providing insights into shared characteristics, such as oncogenicity.

To this aim, we develop a two approaches to address a classification task. We start by computing a diverse set of multilayer topological features, focusing on the ones that allow us to statistically distinguish networks sampled from ensembles of oncogenic and non-oncogenic viruses. Then machine learning techniques were exploited to perform a classification between virus–host interaction PPI networks associated to oncogenic and non-oncogenic viruses, and by doing so, they allow also to identify sets of proteins that play a significant role in predicting oncogenic potential.

By applying functional enrichment analysis to this set of pivotal proteins, a noteworthy emphasis emerges on pathways intimately linked to chromatin structure, a phenomenon extensively associated with the onset of cancer as evidenced by numerous studies [45,46,47]. Additionally, we observe a convergence with well-established cancer-related pathways, including the WNT pathway, TP53, and pathways encompassing sumoylation processes, all of which play pivotal roles in our quest to comprehend viral oncogenic mechanisms.

## 2. Materials and Methods

The work is based on the analysis of PPI data sourced from online databases and processed to create models describing the interaction between viruses and host. Successively multiple PPI networks corresponding to different viruses, are combined into a multilayer network framework, creating the expanded dataset to be used in the analysis phase.

### 2.1. PPI Networks as Proxy for Virus–Human Interactions

This study relies on the BIOstring database [48], a large repository of protein–protein interaction (PPI) data that has been curated by integrating information from various sources, including BioGRID [49] and STRING [50,51]. Specifically, the database contains PPI interactions between 80 viruses and human cells, where 8 viruses are categorized as oncogenic and 72 as non-oncogenic. This classification was established on strongly confirmed clinical literature. For both human-human and virus–human PPIs, we chose to consider a threshold equal to 0.7 on the confidence score provided by STRING and BIOGRID. This results in a huge variety of interaction types, including genetic, co-expression, physical, gene neighborhood, gene fusion, gene homology, text mining links, giving our model a broad descriptive view of the underlying systems.

Given that our aim is to construct networks representing physical interactions between proteins in the human cell, it is important to examine the interaction detection methods underlying the links in the BioSTRING dataset. To this end, we analyzed the main sources of interaction evidence in the two datasets used to generate BioSTRING, namely BioGRID and STRING. As reported in Section S0 of the Supplementary Materials, a substantial fraction of interactions in both datasets originates from experimental techniques capable of detecting physical protein–protein interactions, or from curated databases. Therefore, we conclude that the majority of interactions in BioSTRING represent physical contacts, supporting the suitability of these PPI networks to model physical interactions within the human cell.

Following this procedure the human interactome consists in a network of 15,131 nodes and 719,552 edges. The number of virus–host interactions depends on the single virus, and further details can be found in [28]. Note that this data represents a composite, “mean field” model, as the PPIs are collected under diverse conditions and may involve various human cell types.

Concerning virus–host interactions, the core concept is that when a virus protein targets a human protein, the resulting effects can vary [52,53]. These effects may include inhibiting or disrupting certain interactions while creating new ones. Instead of predicting how the human interactome might change, the focus here is on identifying the region of this large network that is most likely to be affected. By studying this specific subset of the whole human interactome, we aim to gain insights into the strategies employed by the virus to attack a human cell.

**Figure 1:**
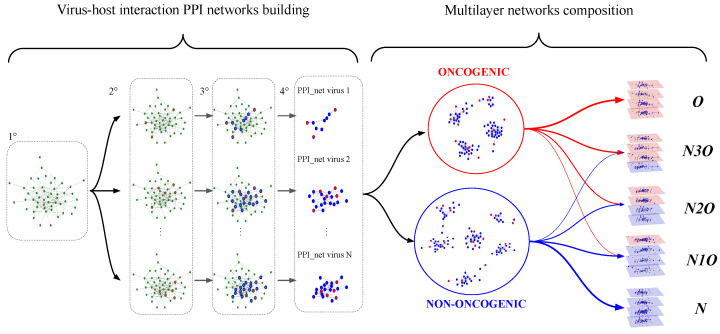
Schematic illustration of the workflow: The figure represents the two main phases of the creation of the analysis dataset. In the first one, depicted in the right side of the figure, an illustrative example demonstrates how the protein–protein interaction (PPI) networks describing the interaction between each virus and the human interactome are built. The first step consists of considering the entire human PPI, built by integrating PPI data from different data sources, represented graphically in the left-most image of the plot, showing a network with green nodes. In the second step, for each virus, the human proteins directly targeted are selected, and they correspond to the nodes highlighted in red in the figure. To expand the number of nodes likely to be influenced by the virus, in the third step, the nearest neighbors (i.e., first-neighbors) of the directly targeted proteins are added, which are highlighted in blue in the figure. Finally, in the fourth step, the human interactome is subset only to the highlighted nodes, resulting in a set of PPI networks, each representing the interaction network between a virus and the host. The second phase consists of building multilayer network samples (right side). In particular, each of the PPI networks created earlier can be classified as being associated with an oncogenic or non-oncogenic virus, based on the a priori information retrieved in the data collection phase. These networks are then combined in groups of 4, considering all possible combinations of oncogenic and non-oncogenic networks to be grouped. Each group of networks is then organized in a multiplex framework, creating a dataset to be used in further analyses.

The construction of the PPI networks associated to each virus is described in the left side of Figure 1 and further details are provided in the Section S1 of Supplementary Materials. At the end of such procedure, we end up having a set of 80 PPI networks, each associated to a specific virus, describing their interactions with the human cell. The name of the viruses used to build the dataset are reported in Table 1.

### 2.2. Combining Multiple Virus–Host Interaction PPI Networks to Uncover Features Shared by Several Viruses

To exploit the full potential of PPI networks, we employed a multilayer framework, which involves combining a fixed number of virus–host interaction PPI networks over the total 80 belonging to our dataset, in a layered structure, in which each layer corresponds to one of the chosen networks. This approach does not discard, a priori, any available information, and it has been used in a variety of disciplines to show that it is often better than considering layers in isolation or aggregating them (see [33] for a recent review).

The multilayer framework serves several key objectives. Firstly, it allows us to interpret each layer as a distinct source of external perturbations–specifically, distinct viral infections–on the human interactome. Secondly, by combining multiple systems (i.e., virus–host PPI networks) that share a common feature, we can identify common properties among these individual systems, which underlie this common feature of interest. Lastly, this framework allows us to generate a wide array of unique samples, built by combining fixed-size sets of virus–host PPI networks. Having generated a high number of these sets, it is possible to ’robustly’ investigate the statistical distributions of various descriptors extracted from these networks.

In this sense, the multilayer representation is particularly advantageous, as it enables the study of multiple virus–host interaction networks within a unified framework, revealing collective properties that emerge only when these systems are analyzed together; by contrast, examining each PPI network in isolation would primarily expose virus-specific features, obscuring the shared signatures linked to oncogenicity that our multilayer approach is designed to uncover.

In practice, each layer nodes correspond to proteins involved at least in one of the virus–host PPI networks included in the multilayer sample, and inter-layer connections link each node to its replicas in the other layers, allowing for the passage of information between layers [54].

Such a procedure is represented graphically in the right side of Figure 1, while mathematical details are provided in Section S2 of Supplementary Materials.

We chose to include 4 virus–host PPI networks in each multilayer network sample, representing a trade-off between the computationally complexity of the resulting object (each virus–host PPI has a number of nodes of order $10^{4}$ and $7\cdot 10^{5}$ edges), and the possibility generate a large number of samples, resulting in combination of virus–host interaction PPI networks associated to different viruses. In fact, the larger the size of such groups, the larger the number of possible combinations. Multilayers with 4 layers appeared to be the best trade-off between these 2 aspects.

As reported before, each virus is classified as belonging to the oncogenic or non-oncogenic class, thus each 4-element combination of virus PPI networks is grouped based on the number of oncogenic and non-oncogenic viruses in it.

In the following, the various combinations of oncogenic and non-oncogenic layers used to create these samples are referred to as “combination sets”. Each combination set is denoted by a label that indicates the number of layers of each type it includes. For simplicity, we also designate layers associated with virus–host interaction PPI networks linked to oncogenic viruses as “oncogenic layers”, and similarly for “non-oncogenic layers”.

Our aim is to study (dis)similarities between descriptive quantities that can be extracted from the different combination sets, thus revealing if the different proportion of oncogenic and non-oncogenic layers can be a statistically discriminating feature over the data. Table 2 reports combination sets considered here, detailing the number of oncogenic and non-oncogenic layers, as well as the total number of possible samples that can be generated from each class, and the corresponding abbreviations used for reference.

### 2.3. Network Features Provide Biological Insights About Virus–Host PPI Systems

The following sections introduce a set of methods for analyzing the topology of multilayer networks that can be linked to biological characteristics of the systems under investigation—in this case, the interactions between viruses and human host cells. These methods aim to reveal insights into how virus–host interactions influence cellular structures, potentially distinguishing the effects of oncogenic versus non-oncogenic viruses.

#### 2.3.1. Identifying the Core of Nodes Biologically Relevant for Oncogenesis

First, we set out to define a framework to identify core nodes in the multilayer networks, which normally constitute a component. In multilayer networks, the concept of what a component is depends on which specific features of the system should be considered. Importantly, all these definitions represent core structures within the system, making them highly relevant [55]. We studied 3 types of components: the largest connected component (LCC), corresponding to the maximum subset of nodes connected by at least one multilayer path between each other, the largest intersected component (LIC), in which nodes must be connected in all the layers independently, and the largest viable component (LVC), where the set of nodes must be connected by the same path in all the layers independently. In particular, nodes belonging to the LVC are usually considered to be very important because they are crossed by paths which are present simultaneously in all the nodes, and for this reason, it is very likely that they are fundamental to determine the overall system behavior [56,57,58].

By studying how these cores change across combination sets, we gain quantitative insights about how different viruses interact with the same regions of the human interactome. Here, we focus on investigating the size of such components, and statistically comparing them to draw conclusions.

#### 2.3.2. Robustness of the Human Interactome to Viral Targeted Attacks

Another significant feature that can extracted from each network is its critical point in response to a targeted percolation process (see [59] for a recent review). In the context of network science, percolation can be interpreted as a way to simulate an attack process aimed at network dismantling. Here, we use the multilayer pagerank versatility [32,60] to rank the nodes to be used for targeted attacks (see Section S2 of Supplementary Materials for further details). From a biological perspective, this quantity identifies the proteins that attract the majority of information flows (under the assumption of a diffusive process), indicating potential functional relevance.

The critical point corresponds to the minimum fraction of nodes to be attacked to disintegrate the largest connected component of the system. Therefore, it provides insights into the network’s responsiveness to external disturbances and, in the context of this study, it is related to how vulnerable the virus-influenced region of the interactome is to external perturbations. Observing the size of the largest and second-largest components of the network as nodes are removed allows us to identify the fraction of removed nodes at which a phase transition occurs. This transition marks the shift from an ordered phase, where a dominant, well-connected component governs the system, to a disordered phase, where no single dominant cluster of connected nodes exists.

#### 2.3.3. Modular Structure Is Related to Oncogenicity

The final set of features under analysis are those extracted from community detection [61,62], a method focused on identifying groups of nodes that are organized at the mesoscale, according to some prescription, from modularity maximization [63,64] to inferring the parameters of a generative model such as the degree-corrected stochastic block model (DCSBM) by means of a Bayesian approach [65]. In this work we use the second method, that has been also reliably extended to multiplex networks like the ones we have built [66]. In our study, this analysis offers insights into the network’s dispersion: the greater the number of communities within the system, the more likely it is that a higher number of functions are associated with the set of nodes in the network, indicating higher functional differentiation and consequently a more prominent systemic behavior. Conversely, a smaller number of communities might suggest that most nodes are linked to similar functions. Of course, this is true under the hypothesis that groups of proteins corresponds to modules with special structural or functional meaning, e.g., specific biological processes within a cell. This is true if we further assume that these interactome subsets we are considering are similar to the original full interactome, in which network communities correspond to biological processes or genes involved in the same functions [11,67].

Another quantity of interest is modularity [63]: the higher the modularity, the more segregated the communities are, suggesting fewer connections between them and a more marked group mesoscale structure.

#### 2.3.4. Exploration of Combination Sets Based on Dimensionality Reduction of Topological Parameters

After analyzing each of the considered features separately, as described in the previous paragraphs, the objective is to combine these different quantities to check if even with this method it is possible to propose a strategy to distinguish samples belonging to the different combination sets. To accomplish this, the UMAP [68] algorithm, a dimensionality reduction technique centered on unsupervised clustering, is employed. UMAP aims to maintain the global structure of high-dimensional data while reducing it to a lower-dimensional space. It was chosen between the other analogous algorithms for its ability to detect non-linear relations. Out of the six extracted features, only four were utilized in this phase. As demonstrated earlier, LCC dimension is closely tied to network size and could introduce bias, while the dimensions of LIC and LVC are highly similar, offering redundant information. The final set of features used to differentiate the parameter space includes percolation critical point, LVC dimension, the number of non-empty communities, and modularity for each multilayer network.

Using UMAP, each point is projected onto a two-dimensional space. Subsequently, a support vector machine algorithm with a Gaussian kernel is applied to separate the reduced parameter space into two regions: an “oncogenic region”, associated to samples containing only oncogenic layers, and the “non-oncogenic region” associated to samples composed only by non-oncogenic layers.

#### 2.3.5. Functional Validation of Findings

At this point the following question could arise: are these results coming from a model that actually contains information about the human cell and its interaction with viruses, or just to the connectivity of human proteins in the interactome?

To investigate this further, we compared the results obtained above with results obtained after a randomization procedure applying random rewiring using the configurational model [69] of the complete human interactome, before extracting the virus–host interaction networks. From this we apply analogous analyses to the ones described in the previous section and compare them statistically, looking for significant differences. Such procedure is repeated multiple times to create a representative null model.

### 2.4. Classification of Oncogenic vs. Non-Oncogenic Virus–Host Interaction PPI Networks Using Machine Learning

After proposing a method to distinguish virus–host interaction multilayer networks composed by different number of oncogenic and non-oncogenic layers based on topological features, we aim to explore methods to perform classification of individual viruses. This approach seeks to determine whether a single virus–host interaction PPI network is associated with an oncogenic virus or not. An important secondary objective is to identify key proteins relevant for classification, potentially shedding light on mechanisms underlying virus oncogenicity.

To accomplish this we exploited the power of machine learning classification algorithms, using as inputs the vectors produced from an embedding procedure of the multilayer networks used in the previous section. Such an embedding assigns to each node a value proportional to its multi page-rank versatility, creating a different fixed size vector for each possible sample in the dataset. The pipeline is described in more detail in the Supplementary Material section.

Using the dataset from the previous analysis sections composed of 4-layer multilayer network samples, our chosen strategy to identify the contribution of a single layer associated with a specific virus focuses on classifying samples composed only by oncogenic layers, from the O combination set, from ones containing only one non-oncogenic layer, thus belonging to N1O. This choice follows the fact that by considering such combinations sets, it is possible to have training and test sets large enough to perform a training procedure. If the performance of the classification algorithms is accurate and exhibits good generalization capabilities, it should also possible to propose a classification for PPI networks associated with viruses not used for training by considering them alongside the oncogenic viruses used for the training.

Alongside classification performance, another important requirement for the machine learning algorithm is interpretability. In our case, this means being able to extract the most relevant input features for the classification. This allows us to achieve our secondary goal, which is to extract a set of proteins relevant for distinguishing between oncogenic and non-oncogenic cases. In this context, we chose a perceptron model, as it is a powerful yet easy-to-interpret machine learning algorithm.

To assess the algorithm’s generalization capabilities and ensure results are not influenced by overfitting, the perceptron is trained and tested multiple times across different datasets, producing different final algorithms. Nevertheless, they all share the same classification objective. In each trial, the training set retains samples from the N1O combination set, which contains an oncogenic layer associated with a specific oncogenic virus. Subsequently, the algorithm’s performance is evaluated on this sample set to determine if it can recognize an oncogenic virus, even if it was not part of the training data.

### 2.5. Functional Enrichment Analysis

To quantify to what extent a list of proteins might indicate the involvement of a biological pathway, function or process, we performed functional enrichment analysis comprising Gene Ontology molecular function, biological processes, and cellular components. The tool used is ToppGene (https://toppgene.cchmc.org/, accessed on 25 November 2025), and the *p*-value threshold over which entries were considered to be significant is chosen as $5\cdot 10^{-2}$ considering the FDR correction.

## 3. Results

### 3.1. Network Features Provide Biological Insights About Virus–Host PPI Systems

We build ensembles of samples from different combination sets and, for each one, we calculate a variety of topological features potentially associated to some underlying biological features of the corresponding systems. Therefore, we compare the statistical distributions resulting from different combination sets and search for statistically significant differences.

#### 3.1.1. Identifying the Core of Nodes Biologically Relevant for Oncogenesis

This section presents results from our analysis of core components in multilayer networks to identify biologically relevant nodes linked to oncogenesis.

The first analysis includes the study of the LCC sizes. We found that they are essentially proportional to the network size, thus not providing a relevant feature for classification, which tends to differentiate only between large and small virus–host interaction networks rather than focusing on more distinctive properties (see Section S3 of Supplementary Materials for details).

Conversely, the LIC and LVC offer more valuable insights. They uncover common structures across different layers, i.e., different virus–host interaction patterns, enabling the identification of shared properties across layers. The focus in our case falls in the oncogenic–non-oncogenic composition of the combination set.

**Figure 2:**
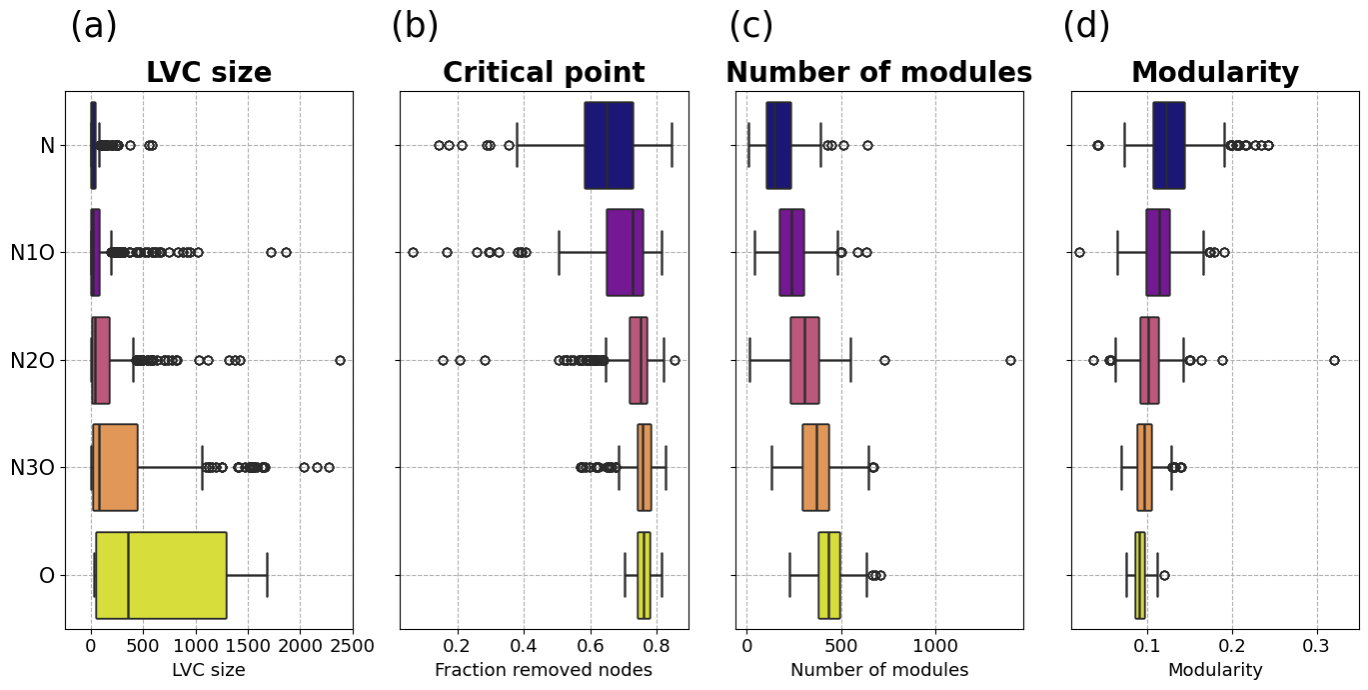
Statistical distribution of the topological features extracted from the multilayer network samples: The figure displays box plots illustrating the distributions of select topological features extracted from the various multilayer network samples within each combination set. For each of those, the colored box represents the first quartile of the distribution, while the line traversing it is the median. The “whiskers” extend to points that lie within 1.5 IQRs of the lower and upper quartile, and then observations that fall outside this range are displayed independently. Specifically, in (**a**), we visualize the distribution of the size of the largest intersected component. Moving on to (**b**), we observe the critical point, denoting the fraction of removed nodes at which the transition phase occurs during node percolation. This transition is determined by following the order indicated by the multi page rank versatility computed for each multilayer network. In (**c**), the number of communities or modules is showcased, while in (**d**), the figure presents the modularity value retrieved with the DCSBM community partition algorithm. Distributions of the extreme cases (O and N) are statistically compared using the Wilcoxon rank-sum test [70]. For LVC size O > N with *p*-value $5\cdot 10^{-15}$; for critical point O > N with *p*-value $9\cdot 10^{-13}$; for number of communities O > N with *p*-value $2\cdot 10^{-13}$; for modularity O < N with *p*-value $2\cdot 10^{-13}$.

Firstly, we studied the distribution of their size, which quantifies how many target nodes are shared by the viruses whose associated PPI networks compose the multilayer network. The statistical distributions of the LIC and LVC sizes are highly similar, often differing by just a few nodes, and thus, in the subsequent analysis, only the LVC case is considered. Figure 2a shows the box plot representing the distributions of the LVC size for each combination set. Notably, in the oncogenic case the distribution of these values skews toward higher values, indicating that oncogenic viruses tend to influence similar and overlapping regions of the human interactome. Statistical analysis show that the distributions associated to the *O* and *N* combination sets are significantly different, yielding a value of $5\cdot 10^{-15}$.

Another crucial finding is that when comparing the O and N3O, the same type of rank-sum test yields a *p*-value of $2\cdot 10^{-3}$, indicating that even inserting just a single non-oncogenic layer into a multilayer network primarily composed of oncogenic virus layers results in a clearly distinguishable distribution from the case of four oncogenic layers.

As an additional analysis to gain deeper insights into the characteristics of oncogenic viruses, we investigate a common core of nodes shared by all the PPI networks associated with them, which on practice corresponds to extracting the LIC of the multilyer built with the eight oncogenic viruses networks. The outcome is a list of 30 proteins extensively reported in Section S10 of Supplementary Materials, highly enriched for pathways involved in transcription, DNA-binding, and sumoylation processes. These include TP53, PARP1, MDM2, MDM4, ABL, and a total of 16 genes that are known to be associated to cancer by comparing them with ones contained in the OncoKB database [71]. An analogous analysis on the N combination set would be less informative because non-oncogenicity corresponds merely to the absence of oncogenicity and is, therefore, unlikely to represent a shared, biologically meaningful feature across viruses.

#### 3.1.2. Robustness of the Human Interactome to Viral Targeted Attacks

This section examines the robustness of the human interactome against viral attacks by analyzing the critical point in a targeted percolation process. By ranking proteins based on their importance in information flow, we assess the network’s vulnerability to disruption, revealing how virus-targeted areas in the interactome respond to external perturbations.

Analyzing the distributions of the node percolation critical points from various combination sets, reported in Figure 2b, reveals a noteworthy trend: a shift towards higher critical point values as the number of oncogenic layers in the combination set increases. Statistically O>N with *p*-value $9\cdot 10^{-13}$.

#### 3.1.3. Modular Structure Is Related to Oncogenicity

This section explores how the modular structure of protein interaction networks relates to oncogenicity by analyzing community structures within multilayer networks. By assessing the number of communities and their modularity, we gain insights into the functional diversity and organizational complexity of the network, which may correlate with the oncogenic potential of the viruses influencing these systems.

The results shown in Figure 2c,d, clearly indicate an inverse relationship between the number of modules and modularity when considering changes in the number of oncogenic layers within the combination set. As the number of oncogenic layers increases, the number of modules significantly rises, while the modularity distributions shift towards lower values. For the number of modules it emerges that O > N with *p*-value $2\cdot 10^{-13}$, while for modularity the opposite happens, i.e., O < N with *p*-value $2\cdot 10^{-13}$.

From these results, it is evident that the presence of oncogenic layers tends to fragment the multilayer networks into a larger number of smaller communities while simultaneously reducing their modularity. This pattern indicates that oncogenicity is associated with a more fragmented and less cohesively organized community structure. Importantly, such a relationship is not trivial in general, as an increase in the number of modules does not necessarily imply a systematic decrease in modularity [63]. In this context, the concurrent rise in the number of modules and drop in modularity suggests a genuine structural disruption, likely reflecting broader alterations in functional organization induced by oncogenic viruses. These findings highlight changes in network modularity and community structure as relevant topological markers of oncogenic potential.

#### 3.1.4. Exploration of Combination Sets Based on Dimensionality Reduction of Topological Parameters

This section focuses on combining topological parameters to distinguish between different combination sets. By applying the UMAP algorithm for dimensionality reduction, we aim to project the selected features into a lower-dimensional space, facilitating the identification of “oncogenic” and “non-oncogenic” regions within the data.

**Figure 3:**
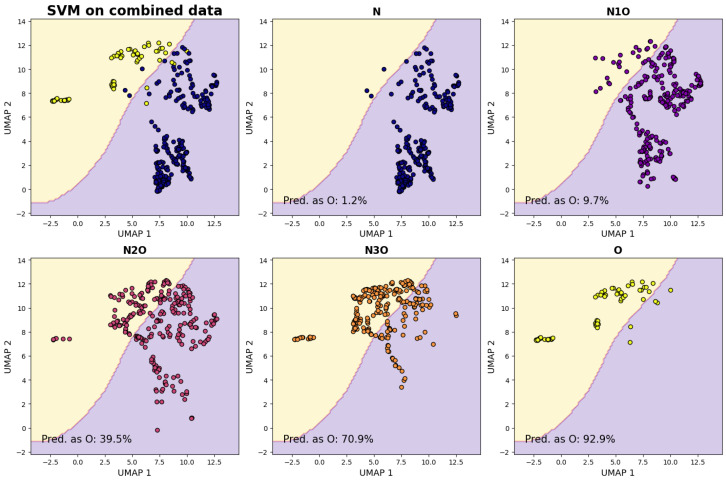
Combined feature classification: Each point in this plot represents a two-dimensional projection generated using UMAP derived from the four-dimensional vectors composed of various topological features linked to multilayer networks within one of the combination sets. These features include LVC size, percolation critical point, number of modules, and modularity. In the top-left plot, points corresponding to samples from category O (yellow circles) and N (blue circles) are presented. The parameter space is effectively divided into two distinct regions, one associated with samples from category O (shaded in yellow) and the other with category N (shaded in blue). This classification is achieved by employing a support vector machine (SVM) with Gaussian kernels. The remaining five plots illustrate how the points generated from different combination sets are distributed within the parameter space. For each of these plots, the fraction of points belonging to the region associated with samples from category O is reported. It becomes evident that this fraction decreases as the number of non-oncogenic layers associated with the combination set increases.

The results are displayed in the top row of Figure 3, and they show a clear distinction between the samples belonging to the two classes. Remarkably, no fine-tuning of the dimensionality reduction algorithms was necessary to achieve such a result.

After the separation of the reduced parameter space in the two regions, we also consider samples generated from the remaining combinations. The four-feature vectors associated to each of their samples is projected into the 2D space using the same UMAP algorithm, falling either in the “oncogenic” or “non-oncogenic” region. In Figure 3 we graphically represent the distribution of the points associated to the samples from each combination set alongside to the fraction of such points falling in the “oncogenic” region. The expectation is that, as the fraction of layers associated with oncogenic viruses increases, the dimensionally reduced points will shift from the “non-oncogenic” to the “oncogenic” region. This expectation is validated by the results depicted in the three images in the bottom row of Figure 3. For each combination set, the fraction of samples classified as belonging to the region associated with the O combination set is also reported, and this value increases as the number of oncogenic layers grows significantly.

The fact that the fraction of samples classified as belonging to the O region exhibits significant change with the ones from N1O, suggest that the algorithm is sensitive to the introduction of a single layer of the non-oncogenic type. It is reasonable to expect that such clear transitions would persist when introducing a new virus, enabling classification through comparison with the known scenario, even in the case of entirely novel viruses.

#### 3.1.5. Functional Validation of Findings

By analyzing the randomized models produced by applying the configurational model to the human interactome network, what we find is that LVCs from randomized models are statistically much less functionally enriched with respect to the one of the original dataset, see Section S5 of Supplementary Materials. The percolation analysis reveals that for the randomized model the networks are statistically less robust, see Section S6 of Supplementary Materials, while the values of the modularity suggests that rewiring the original human interactome, the resulting multilayers composed of virus–host interaction networks are characterized by a weak block structure, see Section S7 of Supplementary Materials. All these findings strengthen the claim that the dataset over which the analysis is performed indeed contains biological relevant information about the relationships between viral proteins and specific human cell processes, and thus, the previous and following analysis can be considered meaningful.

### 3.2. Classification of Oncogenic vs. Non-Oncogenic Virus–Host Interaction PPI Networks Using Machine Learning

This section addresses the classification of virus–host interaction PPI networks to differentiate between oncogenic and non-oncogenic viruses. By using interpretable machine learning algorithms, which in our model correspond to a perceptron, we aim to identify key proteins for classification and uncover mechanisms related to virus oncogenicity.

As reported in Table 3, the performances are strong in nearly all trials, resulting in very high classification precision.

Following the training of the perceptron, it is possible to extract information about relevant input features, in this case, human proteins, which can be associated with classification into the oncogenic or non-oncogenic classes. This is achieved by analyzing the values of the perceptron’s weights. These weights are organized into two sets, each corresponding to a possible output, corresponding to whether the sample contains an oncogenic layer or not. Each set has a number of entries equal to the number of human proteins. The concept is that if the absolute difference between the values of two weights corresponding to the same protein but associated with the two possible outputs is significant, it can be inferred that when a protein is characterized by substantial centrality in the network, it is strongly positively associated with one output and strongly negatively associated with the other.

Employing this method and intersecting the top 200 proteins obtained from each training trial of the perceptron, a list of 81 proteins is generated. By performing a functional enrichment analysis based on Gene Ontology, the results show also in this case a great relevance of processes involved in chromatin structure. The complete list of such proteins and the table depicting the result of the Gene Ontology functional enrichment analysis are reported in the Section S11 of Supplementary Materials.

### 3.3. Specificity on Oncogenic and Non-Oncogenic Classification Task

Viruses can be grouped according to numerous biological, structural, and functional characteristics, and oncogenicity represents only one of many possible classification criteria—often not even the most distinctive. Considering also the relatively large number of parameters of the perceptron model, it is reasonable to question whether the classifier might simply be learning virus-specific signatures unrelated to oncogenicity, and whether similar performance could be achieved when targeting an arbitrary set of viruses.

To test this possibility, we defined a new “target” virus class by selecting viruses at random under the constraint that their virus–host interaction networks have a size distribution comparable to that of oncogenic viruses. These viruses do not share any known biological property and, therefore, do not constitute a meaningful class a priori. Using this new grouping, we constructed multilayer samples containing either zero or one layer corresponding to a randomly selected target virus and trained the perceptron across multiple trials following the same protocol as in the original analysis.

The classifier achieved high accuracy also in this scenario. However, functional enrichment analysis performed on the most informative proteins—extracted as in the oncogenic vs. non-oncogenic case—revealed enriched pathways unrelated to chromatin structure or other processes associated with oncogenicity. This demonstrates that while the machine learning framework is general and capable of distinguishing arbitrary virus sets, the biological signals uncovered in the oncogenic classification task are not generic artifacts of the method. Instead, they reflect meaningful, biologically interpretable patterns specific to oncogenic viruses.

Additional results, including GO functional enrichment analysis for this control experiment, are provided in Sections S8 and S12 of the Supplementary Materials.

## 4. Discussion

Studying oncogenicity in viruses is complex since viruses employ many different mechanisms to infect their hosts. This study aims to gain insights into these topics using a network medicine approach based on protein–protein interaction (PPI) networks, with the goal of offering valuable information for ongoing medical research on viral oncogenicity.

The multilayer framework has shown significant potential for this analysis, enabling precise and quantitative descriptions of the impact that specific viruses can have on the human interactome. Topological analysis reveals clear statistical differences between samples obtained from oncogenic and non-oncogenic virus PPI networks. This distinction is further supported by the combined feature analysis, where the separation between the two categories becomes even more pronounced.

Focusing on the distribution of the largest intersected component (LIC) and largest viable component (LVC) dimensions, higher values associated with oncogenic samples suggest that oncogenic viruses generally target human proteomic regions that are more similar to each other compared to non-oncogenic viruses. This may be linked to specific mechanisms associated with oncogenicity. To explore this possibility, we extracted the set of nodes that are connected in all the PPI networks associated with oncogenic viruses. Subsequently, we performed an enrichment analysis with Gene Ontology, considering molecular functions, biological processes and cellular components. Interestingly, many results are related to proteins involved in chromatin structure, a fundamental aspect of cell life, which influences many of its processes [72]. In particular, alterations in chromatin structure have been found to contribute to the development and progression of cancer [45]. Various processes accompany alterations in chromatin organization in cancer cells, including changes in DNA methylation patterns, histone modifications, and higher-order chromatin folding [46,73].

One key aspect is the epigenetic regulation of gene expression through modifications of chromatin structure. Epigenetic modifications, including DNA methylation and histone modifications, can lead to the silencing or activation of specific genes involved in cancer-related processes such as cell proliferation, differentiation, and metastasis.

Several studies have focused on the characteristics of viruses that were found to be oncogenic [74,75]. The main pathways targeted are quite consistent amongst different types of oncogenic viruses and they include important regulatory factors that we also identified as characteristic in this study such as TP53, PARP, and others.

Considering the distribution of the percolation critical point, a statistically significant difference between oncogenic viruses and others is evident. Specifically, samples composed of oncogenic virus layers tend to have a higher associated critical point, indicating that the system is more robust against targeted attacks. This implies that this kind of viruses may target areas of the human interactome that are more robustly connected, potentially because they belong to more important and structured processes within human cells, likely of earlier evolutionary origin and relevant to a broader number of cell types.

It was recently noted that oncogenic transformation might re-activate primarily genes of ancient evolutionary origin, more involved in fundamental cellular processes that have been conserved since unicellular organisms [76,77]. These genes are broadly expressed and they are associated with growth and other pathways that are less needed in fully differentiated normal cells in the context of tissues in multi-cellular organisms.

As for quantities related to the community partition of the system, a marked difference exists between oncogenic and non-oncogenic cases. The oncogenic case is characterized by a larger number of modules and lower modularity than the non-oncogenic case. This suggests that it may be more challenging to identify clear community structures in oncogenic systems due to a more homogeneous distribution of links between nodes, an effect that could be related to a more systemic nature of oncongenic viruses.

While each of these measures offers insights into the separation of the two virus categories, no clear, easily identifiable threshold between them is observed. To address this, we propose integrating different features to improve the classification procedure. UMAP visualization effectively illustrates this, with a clear separation between oncogenic and non-oncogenic regions. As the number of oncogenic layers increases, the distribution shifts from the oncogenic region to the non-oncogenic one, enforcing the confidence in the classification results.

It is well known that the data from which the models used for the analysis are built are characterized by a huge degree of uncertainty and potentially huge biases due to experimental reasons [78,79]. For this reason we tested our results by randomizing the model, which consisted in performing random rewiring of the complete human interactome from which each time the virus–host interaction networks were extracted. Similar analysis to the ones described above were performed to check for statistical differences that could support the assumption according to which the original data can indeed form a model able to well describe some aspect of the human cell biology. The complete results of such analysis are reported in Sections S4–S7 of Supplementary Materials, and they all lead to the conclusion that the randomized models indeed do not provide highly relevant biological results, reinforcing the results reported in the article.

The next step involves considering a global description of each network which is used to propose a method to perform classification with the ultimate goal to isolate the contribution of the single layer, and at the same time also being able to keep track of the relevance of the single nodes. We introduced a machine learning pipeline based on the perceptron architecture, which allows for the extraction of a set of relevant nodes for classification by analyzing the weights, as detailed in the results section. By performing GO functional enrichment analysis over this set, we obtain results very similar to the ones obtained from the largest components analysis, consistent with our initial findings.

Upon completing perceptron training with different datasets, we apply a procedure to extract the most relevant input features for classification. The top 200 proteins from each trial are intersected to obtain a core set of 81 key proteins. We then use this core to perform a Gene Ontology molecular function enrichment analysis to explore potential associations between these proteins and cancer. The results reveal a high relevance of processes involved in chromatin structure, confirming the insights coming from the previous analysis.

A critical consideration is the potential presence of biases that could impact the final classification results. Viruses can be classified upon various characteristics, such as the type of nucleic acid they contain (DNA or RNA), the type of infection they cause in the body (intestinal, respiratory…), size and morphology (helical, icosahedral, prolate, enveloped, …) and taxonomic identification. The categorization of DNA vs. RNA viruses was explored, in particular regarding the results of the machine learning method, as illustrated in Section S9 of Supplementary Materials, and appears not to be a relevant bias factor in the classification task. The other possible differentiation between viruses could be explored in future work, leading to a more robust classification analysis.

## 5. Conclusions

In conclusion, this study demonstrates the potential of a network-based approach to distinguish oncogenic and non-oncogenic viruses by analyzing multilayer PPI networks. By leveraging topological network features and machine learning models, we were able to perform a meaningful classification between viruses belonging to the two classes and, at the same time, gaining insights into potential biological factors related to viral oncogenicity. These results highlight that proteins involved in transcription regulation, sumoylation, and chromatin structure are significantly enriched, indicating that viral oncogenicity is closely linked to the manipulation of host gene expression and protein stability pathways.

For future work, one key improvement lies in refining the virus–host PPI network construction. Rather than simply sub-setting the human PPI network to the first neighbors of virus-targeted proteins, future approaches could integrate a broader biological context, including indirect interaction pathways, structural proximity, or additional data sources to achieve a more accurate representation of viral influence on host cellular processes. Another potential enhancement involves quantifying oncogenicity as a spectrum rather than a binary classification, which could improve sensitivity to partially oncogenic viruses. Further, exploring virus characteristics—such as infection type or taxonomic data—could make the classification model more robust and biologically informative.

Overall, our findings suggest that it is possible to employ a procedure to inspect possible long term effects of viruses on humans without using data coming from clinical research. This can be applied to novel viruses, providing as example some insights that could help to orientate clinical research.

## Figures and Tables

**Table 1:** Viral species included in the curated virus–human protein–protein interaction (PPI) dataset: The table organizes all viruses considered in this study according to genome type (RNA or DNA) and oncogenic potential. These viral species served as the reference set for assembling and harmonizing experimentally supported virus–host PPI networks, forming the basis for all comparative and integrative analyses presented in this work.

NON-ONCOGENIC	
RNA (51)	DNA (21)
Avian_infectious_bronchitis_virus, Avian_leukosis_virus_RSA	African_swine_fever_virus,
Bluetongue_virus_10, Bovine_viral_diarrhea_virus	Bean_golden_yellow_mosaic_virus,
Chikungunya_virus, Cucumber_mosaic_virus	Cottontail_rabbit_papillomavirus,
Dengue_virus_type_1, Dengue_virus_type_2	Equine_herpesvirus_2,
Dengue_virus_type_3, Dengue_virus_type_4	Gallid_herpesvirus_2,
Encephalomyocarditis_virus, Equine_arteritis_virus	Human_adenovirus_C_serotype_2,
Foot-and-mouth_disease_virus, Hantaan_virus	Human_cytomegalovirus,
Hendra_virus, Hepatitis_E_virus_genotype_1	Human_herpesvirus_1,
Human_coronavirus_229E, Human_hepatitis_A_virus_genotype_IB	Human_herpesvirus_2,
Human_immunodef._type_1_group_M_sub._B, Human_immunodef._type_2_subtype_e_A	Human_herpesvirus_6A,
Human_metapneumovirus, Human_parechovirus_2	Human_herpesvirus_6B
Human_respiratory_syncytial_virus_B, Human_rhinovirus_A_serotype_89	Myxoma_virus,
Human_SARS_coronavirus, Human_SARS_coronavirus_2	Murine_minute_virus,
Influenza_A_virus, Influenza_B_virus	Simian_virus_40,
Japanese_encephalitis_virus, Marburg_virus	Swinepox_virus,
Measles_virus, Mumps_virus Vaccinia_virus,	Varicella-zoster_virus,
Moloney_murine_leukemia_virus, Murine_coronavirus	Variola_virus,
Newcastle_disease_virus, Norwalk_virus	Yaba_monkey_tumor_virus
Poliovirus_type_1, Potato_mop-top_virus,	Murid_herpesvirus_1,
Potato_virus_Y, Rabbit_hemorrhagic_disease_virus	
Rift_valley_fever_virus, Rotavirus_A	
Salivirus_A, Semliki_forest_virus	
Sendai_virus, Simian_immunodeficiency_virus	
Sindbis_virus, Tomato_spotted_wilt_virus	
West_Nile_virus, Yellow_fever_virus	
Zaire_ebolavirus	
**ONCOGENIC**	
**RNA (2)**	**DNA (6)**
Hepatitis_C_virus_genotype_1a,	Epstein–Barr_virus,
Human_T-cell_leukemia_virus_1	Hepatitis_B_virus_genotype_C_subtype_ayr,
	Human_herpesvirus_8_type_P,
	Human_papillomavirus_type_16,
	Human_papillomavirus_type_18,
	Human_papillomavirus_type_5

**Table 2:** Combination sets used in this study, built from combining oncogenic and non-oncogenic layers. The number of possible different samples that can be obtained from the data is reported in the last column.

Acronym	Layers Combination	Number of Possible Combinations
O	4 oncogenic	70
N	4 non-oncogenic	1,028,790
N1O	3 non-oncogenic + 1 oncogenic	477,120
N2O	2 non-oncogenic + 2 oncogenic	71,568
N3O	1 non-oncogenic + 3 oncogenic	4032

**Table 3:** The table presents the performance results of individual perceptron models trained using datasets in which samples containing specific oncogenic viruses were excluded, specifically the training and validation accuracy at the end of each model training. The OncoTest pred column contains the accuracy values of the predictions performed over the samples containing the excluded oncogenic virus PPI layer.

Trial Name	Excluded Onco Virus	Train Acc	Val Acc	OncoTest Pred
EB	Epstein–Barr	0.923	0.931	0.962
HBC	Hepatitis B gen. C, ayr	0.916	0.9204	0.670
HC1	Hepatitis C gen. 1a	0.931	0.920	0.886
HV8P	Hum. herpesvirus 8 type P	0.923	0.898	0.969
PV16	Hum. papillomavirus type 16	0.918	0.911	0.736
PV18	Hum. papillomavirus type 18	0.917	0.916	0.833
PV5	Hum. papillomavirus type 5	0.932	0.932	0.402
TL1	Hum. T-cell leukemia 1	0.915	0.920	0.860

## Data Availability

This study utilizes the dataset described in [48], where additional information and resources can be found. The Jupyter notebooks used for the analysis and figure production can be found in https://github.com/francescozambelli/OncoVirus (accessed on 25 November 2025).

## Supplementary Materials

The following supporting information can be downloaded at: https://www.mdpi.com/article/10.3390/e27121248/s1. S1: Mathematical model for virus–host interaction networks. S2: Multilayer networks formalism and multi-pagerank versatility S3: Components analysis, LCC. S4: Comparison with null model. S5: Comparison with null model, LVC. S6: Comparison with null model, percolation. S7: Comparison with null model, community partition. S8: Machine Learning cross-checking. S9: DNA vs RNA viruses. S10: GO enrichment analysis results, common nodes between all oncogenic layers. S11: GO enrichment analysis results, highly relevant proteins from perceptron weights analysis. S12: GO enrichment analysis results, highly relevant proteins from perceptron weights analysis with different target set.

## Abbreviations

The following abbreviations are used in this manuscript:

PPI	Protein–protein interactions
LCC	Largest connected component
LIC	Largest intersected component
LVC	Largest viable component
GO	Gene Ontology
